# Hematopoietic Stem Cell Transplantation in Refractory Crohn’s Disease: Should It Be Considered?

**DOI:** 10.3390/cells11213463

**Published:** 2022-11-02

**Authors:** Simon Reider, Lukas Binder, Stefan Fürst, Stefan Hatzl, Andreas Blesl

**Affiliations:** 1 Christian Doppler Laboratory for Mucosal Immunology, Johannes Kepler University Linz, 4020 Linz, Austria; 2 Department of Internal Medicine 2 (Gastroenterology and Hepatology), Faculty of Medicine, Kepler University Hospital, Johannes Kepler University, 4020 Linz, Austria; 3 Division of Gastroenterology and Hepatology, Department of Internal Medicine, Medical University of Graz, 8036 Graz, Austria; 4 Intensive Care Unit, Department of Internal Medicine, Medical University of Graz, 8036 Graz, Austria; 5 Division of Hematology, Department of Internal Medicine, Medical University of Graz, 8036 Graz, Austria

**Keywords:** Crohn’s disease, inflammatory bowel disease, hematopoietic stem cell transplantation, chemotherapy

## Abstract

Hematopoietic stem cell transplantation (HSCT) is widely used in benign and malignant hematological diseases. During the last decade, HSCT, mainly autologous, also gained increasing attention in the treatment of refractory autoimmune diseases. Crohn’s disease (CD) is an inflammatory bowel disease leading to transmural inflammation potentially affecting all parts of the luminal gastrointestinal tract. Despite improving therapeutic options, including various biologics, some patients are refractory to all lines of available conservative therapy, leading to increased morbidity and reduced quality of life. Apart from surgery, HSCT might be a reasonable treatment alternative for refractory CD patients. This review aims to describe the current role of HSCT in CD and discusses the procedure, the correct patient selection, the clinical efficacy from initial remission to following relapse rates, and complications of this treatment.

## 1. Introduction

### 1.1. Crohn’s Disease

Crohn’s disease (CD) is an inflammatory bowel disease (IBD) that can cause transmural inflammation in all parts of the luminal gastrointestinal tract. Most commonly, the small intestine, especially the terminal ileum, and the colon are affected. CD leads to mucosal swelling and ulcers and in severe cases may exhibit a stricturing and penetrating phenotype, causing stenosis or fistulas. These complications can be associated with life-threatening conditions such as bacteremia and sepsis through infections or acute intestinal obstruction requiring surgical interventions. CD is further associated with extraintestinal manifestations involving joints, eyes, or skin, which may represent a significant burden for patients [1]. The etiology of the disease is still incompletely understood. However, a multifactorial genesis involving genetic susceptibility, alterations of the gut microbiome, and environmental triggers that ultimately lead to a disturbed host immune response is the commonly accepted model [2]. Increasingly, IBDs are perceived as a spectrum of disease with different ratios of contributions by genetic and environmental factors. These range from almost exclusively genetically driven disease (e.g., monogenic forms of IBD, such as those associated with mutations in IL10RA/B or the X-linked inhibitor of apoptosis (XIAP) [3] and entities with a large genetic contribution such as very early onset (VEO)-IBD) [4], to phenotypes that are more dependent on environmental factors. In general, disease based on a high genetic risk burden and monogenic disease tends to manifest early in life and is associated with a higher risk of treatment failure, as the underlying pro-inflammatory processes tend to be more resistant to therapeutic intervention [5].

To date, there is no known cure for CD. Treatment strategies focus on improving the patient’s symptoms, reducing intestinal inflammation to achieve clinical remission, as well as endoscopic, histologic, and transmural healing and avoiding severe complications [6].

A variety of therapeutic options for CD have been introduced over the last decades. These range from local anti-inflammatory agents to immunosuppressants and specific monoclonal antibodies, which target elements of the inflammatory cascade involved in unregulated intestinal inflammation [7]. While the therapeutic options are expanding, rates of remission observed in large phase III clinical trials of new substances are usually reported to be around 30–50% [8,9,10,11,12], further supporting the notion of IBD as a disease spectrum with different molecular pathways driving inflammation. This ultimately results in variable and currently not predictable response to the available therapeutic options [13].

### 1.2. Hematopoietic Stem Cell Transplantation (HSCT)

Hematopoietic stem cell transplantation (HSCT) was initially established as treatment for malignant hematological diseases such as leukemia and malignant lymphoma. Since the first attempts in the late 1930s, HSCT has emerged as a feasible treatment option in a broad range of benign and malignant applications [14].

HSCT mainly comprises two different procedures: (1) autologous stem cell transplantation—the source of the stem cells is the patient himself—and (2) allogeneic stem cell transplantation—the source of stem cells is a healthy related or unrelated person with immunologic compatibility. Hematopoietic stem cells can be obtained from different sources (e.g., peripheral blood, bone marrow, and umbilical cord blood) [15].

The procedure of HSCT consists of several steps ranging from stem cell mobilization by the support of granulocyte colony stimulating factor (G-CSF) to stem cell collection and conditioning with high-dose chemotherapy, including agents such as anti-thymocyte globulin (ATG) and cyclophosphamide to achieve depletion of the autoreactive immunologic memory (T and B cells) before transplantation. These conditioning regimens vary depending on the condition leading to transplantation and the transplantation protocol. In the state of a maximally depleted immunological memory, HSCT is performed to reconstitute a distinct immune system and to re-establish immune tolerance [16].

Still, the most common indications for HSCT are hematological disorders such as multiple myeloma, non-Hodgkin lymphoma, Hodgkin lymphoma, and leukemia. However, after half a century in which the procedure was solely used in the domains of hematology and oncology, HSCT started to also emerge as a therapeutic option in severe cases of autoimmune diseases refractory to medical treatment [17]. The most extensively investigated diseases are multiple sclerosis, systemic sclerosis, and CD [18]. However, due to the high treatment-related mortality and post-interventional morbidity exhibited during allogenic stem cell transplantation caused by complex immunologic phenomena, this procedure is widely viewed as unjustifiable for non-malignant diseases such as CD [19]. Therefore, to date, autologous HSCT is mainly used for the treatment of autoimmune diseases.

In CD, HSCT represents an unconventional treatment approach with the potential to reset dysbalanced immune reactions that arise from multiple environmental hits on the basis of genetic susceptibility. This is achieved by potent abrogation of immune cells during induction, eliminating aberrant auto-reactive inflammation. By transplanting hematopoietic stem cells, the immune system is reconstituted, and control of immune reactions is re-established. HSCT impacts all components of the immune system and has broad effects on pro- and anti-inflammatory pathways alike. These include depletion of autoreactive memory B cells and resetting of the T cell receptor repertoire, as well as promotion of regulatory responses [20].

In this review, we discuss HSCT for the treatment of refractory CD focusing on available evidence and highlighting the correct patient selection, procedural steps, and associated side effects, as well as future perspectives.

## 2. HSCT for Crohn’s Disease

### 2.1. First Evidence

The idea of using HSCT in patients with refractory CD emerged from case reports/series during the 1990s reporting improvement or remission of CD in patients receiving HSCT for malignant hematological indications [21,22,23,24,25]. These reports used autologous, as well as allogenic, HSCT. In a case series of six patients suffering from leukemia and CD receiving allogenic HSCT, four out of five patients with active CD before transplantation remained in remission within 4.5 to 15 years. Four patients had HLA-identical siblings as donors, one patient had a related mismatched donor, and one patient an unrelated HLA-matched donor. Five out of the six patients developed acute, and four out of six patients chronic graft-versus-host disease (GvHD). One death due to septicemia occurred three months after transplantation [25]. A retrospective analysis of 11 IBD patients (seven CD, four ulcerative colitis) treated with allogenic HSCT due to acute and chronic myeloid leukemia and myelodysplastic syndrome included 10 patients with HLA-identical donors and one patient with an unrelated mismatched donor. One death due to pulmonary fungal infection was reported and eight patients developed acute GvHD. A total of 9 out of 10 alive patients maintained clinical remission of their IBD within a heterogeneous follow-up time of three months to nearly 10 years [22].

### 2.2. Patient Selection for HSCT in CD

When thinking about the usage of HSCT exclusively for the treatment of CD, one has to define a suitable patient collective. With the emerging availability of a wider range of biologics with a favorable safety profile and the options of modern minimally invasive surgery techniques, indications have to be very rigorous, and the success of the method is also determined by the selection of the right patients. First, it should be ensured that the diagnosis of CD is established. Second, the patient should suffer from a complicated disease course and should have inadequate response to available immunosuppressive and biologic therapies. Third, active disease should be confirmed with biomarkers, endoscopy, and cross-sectional imaging and should carefully be differentiated from irreversible fibrotic strictures. Fourth, infectious complications such as intraabdominal abscesses should be excluded, and the presence of perianal disease should be considered as risk factor for complications of HSCT. Fifth, surgery as an alternative treatment strategy should have been judged as not suited due to risk of short bowel syndrome, technical aspects due to multiple previous surgeries, or because of a definitive refusal of the patient [26,27]. Furthermore, the general condition of the patient must be considered. Chronic active CD predisposes patients to malnutrition and sarcopenia, which was shown to increase mortality in patients receiving allogenic HSCT [28,29]. Additionally, patients should be screened for pulmonary and cardiovascular comorbidities with pulmonary function testing, electrocardiography, and echocardiography, as cardio-pulmonary impairments also propagate the risk for complications of HSCT [30,31].

### 2.3. Technical Aspects of Autologous HSCT for CD

**Figure 1:**
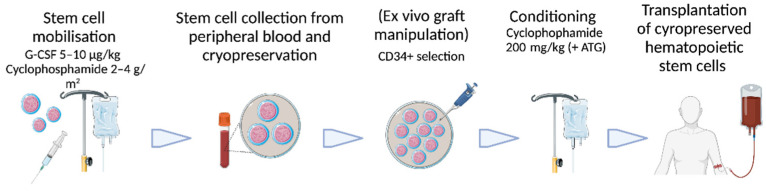
Procedural steps of autologous hematopoietic stem cell transplantation in autoimmune diseases with used drug dosing in studies for Crohn’s disease. G-CSF = granulocyte colony-stimulating factor; ATG = anti-thymocyte globulin. Figure created with Biorender.com and Smart Servier Medical Art. Accessed on 9 September 2022.

As previously highlighted, autologous HSCT is preferred over allogeneic HSCT for the treatment of autoimmune diseases due to the less complicated procedure [32,33]. Autologous HSCT consists of several steps (Figure 1): First, stem cells have to be harvested from the patient. In autoimmune diseases, it is established to use peripheral blood stem cells (PBSC) mobilized by G-CSF. As disease flares have been reported during G-CSF treatment in patients with multiple sclerosis, rheumatoid arthritis, and systemic lupus erythematosus [34,35,36], cyclophosphamide with a dose of 2–4 g/m^2^ is applied before G-CSF treatment with 5–10 µg/kg [27,35]. This regime was also used in the reported trials of autologous HSCT for CD [37,38,39,40,41,42,43,44,45,46,47,48]. To avoid combined immunosuppression, immunomodulators and biologics for the treatment of CD should be discontinued as early as possible before cyclophosphamide application to reduce the risk of infections and to prevent interference with PBSC mobilization. The minimum yield of hematopoietic stem cells (CD34+ cells) is 2 × 10^6^/kg CD34+ cells to grant sufficient engraftment after high-dose chemotherapy, as suggested by the European Group for Blood and Marrow Transplantation (EBMT) [49].

In a second step, the patient has to be prepared for HSCT using a conditioning regime to achieve myelosuppression to consequently enable a reset of the bone marrow after transplantation. Conditioning regimes used in CD patients consist almost uniformly of a combination of cyclophosphamide 200 mg/kg and rabbit or horse ATG. Only one study used cyclophosphamide monotherapy [41]. This is in line with EBMT guidelines suggesting intermediate-intensity conditioning regimens over high-intensity regimes in patients receiving HSCT for autoimmune diseases [49]. A more recent pilot study by Burt et al. investigating non-myeloablative allogeneic HSCT in CD chose a conditioning regime combining cyclophosphamide with fludarabine and alemtuzumab [50].

Before transplantation, the question arises whether to perform ex vivo graft manipulation using CD34+ selection. In CD, nearly half of studies used CD34+ selection, although a clear benefit has not been demonstrated yet in autoimmune diseases, and EBMT guidelines suggest against routine usage of selection outside of experimental settings [27,49]. Additionally, graft manipulation is costly and may require an increased number of CD34+ cells, which have to be obtained during the mobilization process.

### 2.4. Actual Evidence for Autologous HSCT in CD

The evidence of HSCT in CD is mainly limited to relatively small prospective, single-arm, phase I/II studies, retrospective analyses, and case series or even single-patient case reports. The ASTIC trial is to date the only available randomized controlled trial [45,46]. Except for a recent pilot study [50], all studies investigated autologous HSCT (Table 1). CD is the third most common indication for HSCT in autoimmune diseases [51].

First evidence for autologous HSCT to exclusively treat CD dates back to 2003 and 2004. The first report from Burt et al. [53] included two patients, a 22-year-old woman and a 16-year-old boy, with a Crohn Disease Activity Index (CDAI) higher than 250 and severe inflammation on colonoscopy despite ongoing anti-TNF therapy. After HSCT, both patients could achieve clinical remission, could discontinue CD medications, and endoscopic evaluation six months after transplantation showed marked improvement of mucosal inflammation. From Chicago, four patients were reported in 2003 [53]. All of these patients experienced normalization of CDAI after HSCT, despite partly persisting inflammation on colonoscopy. Follow-up was too short to conclude about the ability to maintain remission. Another early case report from Italy was published in 2004 describing a single patient achieving clinical remission and endoscopic improvement after autologous HSCT [37]. The first larger cohort reported came from Chicago in 2010 and was also a first phase I/II study evaluating HSCT in CD, but without a control group and without randomization [38]. Although initial results were encouraging, with all 24 patients achieving clinical remission with a CDAI < 150 after HSCT, relapse rates after five years of follow-up, defined as the need for CD treatment, were 81%. Anyway, roughly half of patients relapsing achieved remission with established CD treatments again. These high relapse rates were confirmed in further reports from the Netherlands, Germany, and UK [40,41,42].

The first, and to date only, randomized, controlled, multi-center trial evaluating HSCT for CD is the ASTIC trial [45] conducted in 11 European transplant centers in six countries. Included patients were adults younger than 50 years suffering from refractory CD who had to have at least three unsuccessful treatment attempts with immunomodulators or biologics. Furthermore, they had been considered not suitable for surgical treatment. The study had a parallel group design, with all patients receiving mobilization with cyclophosphamide and G-CSF. One group was consequently assigned to immediate autologous HSCT and the other to HSCT after one year with conventional care within the first year. During follow-up, approved immunosuppressive/biologic treatments for CD were allowed in both groups. The primary endpoint called sustained disease remission was evaluated after 12 months and included a CDAI < 150 for at least 3 months, no additional active CD treatment within the last 3 months, and no erosion or ulceration in the gastrointestinal tract upon endoscopy. A total of 48 patients received mobilization, 45 were randomized, and 23 finally received HSCT. The primary endpoint, aiming to show the benefit of HSCT, was not met, as only two patients after HSCT reached sustained disease remission compared to one patient in the standard treatment arm. This study was also the only one evaluating HSCT in CD included in a recent Cochrane review on stem cell transplantation in CD [54], as the other six included studies assessed mesenchymal stem cell transplantation for perianal fistulas. Except for a high risk of bias concerning the missing blinding of patients, clinicians, and investigators, the authors of this review judged the study to be at low risk of bias.

The ASTIC trial was re-analyzed with data from 38 patients (23 patients initially randomized to HSCT, 17 patients receiving delayed HSCT after one year, one death, one drop-out) with modified endpoints [46]. The combined primary endpoint of clinical remission with a CDAI < 150 and no corticosteroids for at least three months was achieved by 38% of patients after one year, and endoscopic healing by half of patients. A lower CDAI and shorter disease duration at baseline were associated with reaching the primary endpoint.

In a cohort from Barcelona [44] with 29 transplanted patients, the probability of clinical and endoscopic relapse-free survival (CDAI < 150, SES-CD < 7) was only 15% after five years. More than 50% of patients had relapsed after a median of one year. Also in this study, the majority of patients achieved clinical remission again with consequently initiated anti-TNF therapy, but three patients failed to respond to biologic therapy and required surgery. It is encouraging that clinical remission was finally achieved by nearly all patients with an enhanced follow-up of 3–5 years with different combinations of HSCT, surgery, and biologic therapy. High relapse rates of 73% after three years were also reported in a retrospective evaluation including 82 CD patients from the EBMT database who underwent autologous HSCT [47].

### 2.5. Actual Evidence for Allogenic HSCT in CD

Due to the reported high relapse rates of autologous HSCT and the published promising case series using allogenic HSCT for the treatment of CD concomitant with hematological malignancies [22,25], Burt et al. investigated non-myeloablative allogeneic HSCT for the first time to exclusively treat CD [50]. Nine patients with refractory CD were included in the study. In three patients, unselected matched sibling PBSC was used, and in six patients, umbilical cord blood was used. One death occurred during the study period, but the remaining eight patients were followed for five years. All of these patients remained disease-free; MRI and colonoscopy results between three and five years after transplantation were available in six patients and did not show any signs of disease relapse. Patients receiving HLA-matched sibling transplants had donor T cell engraftment, whereas patients receiving umbilical cord blood did not have engraftment, but this did not seem to have an impact on the efficacy of this method.

### 2.6. Safety of HSCT in CD

HSCT is an intervention with relevant morbidity and mortality [55,56,57,58]. Although autologous HSCT does not impose some risks associated with allogeneic HSCT such as GvHD, it may still cause major complications and mortality in all steps of the procedure (mobilization, conditioning, transplantation, and post-transplantation phase). Febrile neutropenia commonly occurs after mobilization and with high certainty after conditioning [43], potentially leading to bloodstream infections and sepsis. Bacteremia including isolates of *Staphylococcus epidermidis*, *Staphylococcus aureus*, *Echerichia coli*, *Klebsiella pneumoniae*, *Klebsiella oxytoca*, and *Enterococcus faecium* was commonly reported in HSCT trials for CD [37,43,45]. In the ASTIC trial, eight cases of neutropenic sepsis were reported [45]; in the Barcelona cohort, three septic shocks were observed [43]. The ASTIC trial reported a higher rate of adverse events in the HSCT group compared to the control cohort within the first 100 days after conditioning and transplantation [45]. The earlier mentioned recent Cochrane Review about stem cell transplantation for CD did not find an increased risk of adverse events in patients undergoing transplantation, but an indication of an increase in serious adverse events, although certainty of evidence was reported to be low [54].

As mentioned, allogenic HSCT imposes a relevant risk of GvHD. In the early case reports of allogenic HSCT for CD and hematological malignancies, acute GvHD was reported in more than 80% of patients, although most were classified as Grade I or II [22,25]. With the allogenic, non-myeloablative regime used by Burt et al. for CD [50], acute GvHD was not observed, and only one patient developed chronic, limited disease. However, due to the limited number of participants included in this trial, a correct estimation of GvHD is not possible.

Due to the high treatment-related mortality and morbidity of HSCT, instruments for the estimation of HSCT risk are crucial in the decision process regarding whether a patient should be allocated to this procedure. The most commonly used risk prediction tool in allogenic stem cell transplantation is the hematopoietic cell transplantation comorbidity index (HCT-CI), which combines different factors influencing HSCT outcomes in a simple score [59]. The HCT-CI provides valid and reliable scoring of pre-transplant comorbidities that predict non-relapse mortality and survival and could thereby be translated into a non-malignant application of HSCT. Besides the clinical condition of the patient, the donor-specific properties are mainly responsible for graft function and prevention of GvHD. The main criterion for donor selection is HLA compatibility. In unrelated donor selection, matching for the classical high-expression loci (HLA-A, HLA-B, HLA-C, HLA-DRB1) is still the paramount criterion. If more than one equally suitable donors are available, further parameters such as DPB1 epitope matching, blood group, and donor sex may be considered [60]. HLA factors also influence the success of haploidentical transplantation. HLA-DRB1 and -DPB1 mismatching and HLA-C, -B leader, and -DQB1 matching have shown favorable outcomes in patients undergoing haploidentical stem cell transplantation with post transplantation cyclophosphamide therapy [61].

Furthermore, mortality was reported in studies concerning HSCT in CD. In the ASTIC trial, one of the 23 transplanted patients died due to sinusoidal obstructive syndrome 20 days after conditioning. Another death occurred in the Barcelona cohort [43], where a patient died eight weeks after transplantation due to CMV infection leading to multi-organ failure. The third death reported happened in the newest pilot study using non-myeloablative, allogeneic HSCT [50]. This patient died due to disseminated adenovirus infection three months after discharge. Due to this death, the trial stopped recruiting patients.

## 3. Discussion

### 3.1. Risks and Benefits of HSCT in CD

HSCT for autoimmune conditions in general, and for CD in particular, is a rarely pursued strategy, and available evidence is limited. It entails laborious pre-procedural workup to adequately assess and select patients and limit the occurrence and impact of potential adverse events. After the procedure, close and ongoing monitoring of patients is required to recognize and manage short- and long-term complications in a timely manner. Furthermore, mobilization and conditioning steps are associated with considerable toxicity. In contrast to malignant diseases, which are the established indications for HSCT, CD generally follows a more benign course with a much better prognosis quo ad vitam [62], although mortality data specifically for severe CD for which HSCT might be considered are not available. As a consequence, the risks and benefits of performing such a high-risk therapeutic procedure have to be carefully weighed before a decision to perform HSCT in CD is made. Furthermore, expertise and routine in both the treatment of severe and complicated CD, as well as in performing HSCT, are restricted to selected centers.

The potential benefits of HSCT can be deducted from the few published studies. These trials have been performed in a very challenging and highly refractory patient collective, with patients having failed multiple lines of therapy [38,45,47]. Nevertheless, initial success rates of autologous HSCT, although reported with great variance in the different trials, seem to be comparable to those seen in phase-two and three trials of the most recently evaluated biologic therapies for CD [45,47], in which clinical remission rates ranging from 40 to nearly 60% after 12 weeks of treatment have been reported in a less pretreated patient collective [12,63]. As autologous HSCT aims to reboot an aberrant immune system and to abrogate auto-reactive T cell clones, it naturally is not suitable to correct underlying genetic defects which are associated with CD pathogenesis. Consequentially, long-term remission of CD after autologous HSCT is rarely achieved, and relapses are common. However, encouraging results from reported case series and the one RCT performed for autologous HSCT provide evidence that the severity of recurrent disease after HSCT is generally mild to moderate and often targetable by biologic therapies, even those the patient previously had been refractory to. This regained responsiveness is partially responsible for the favorable long-term outcomes that have been reported in transplanted patients. Uniquely, and unlike medical therapies, HSCT does not involve long-term maintenance therapy. Patients are subject to a detailed post-procedural surveillance program but do not routinely receive continued anti-inflammatory treatment, which couldbe considered a potential benefit.

Additional to the risk of HSCT-related mortality in CD [45,47,50], the procedure entails considerable morbidity. The risk of infectious complications is almost inevitable due to the severe myelosuppression that is achieved during conditioning. Furthermore, it has been hypothesized that patients with CD might be even more prone to systemic septic complications because of bacterial translocation from the intestine via inflammatory ulcerations. It has been recommended that CD patients undergoing HSCT receive specific and intense prophylactic antibiotic treatment in combination with bowel rest before the procedure [43].

Other potential long-term sequelae of high-dose cyclophosphamide administered during conditioning include a risk of new cancer development, impacts on the cardiovascular, respiratory, and neurological systems, as well as fertility [32]. However, in the Chicago cohort [38], no interval malignancies occurred over a follow-up period of five years. In contrast, Brierley at al. [47] reported five malignancies after autologous HSCT within a median follow-up of 3.5 years.

The best conditioning regime remains to be determined for autologous HSCT in CD. Most studies used the combination of cyclophosphamide and ATG. ATG exerts diverse effects on the immune system, leading to T cell depletion in peripheral lymphoid tissues and blood and apoptosis of B cells [64].The application of ATG entails a risk, although low, for anaphylaxis [27], and it has been reported that secondary autoimmune disorders may arise after using ATG in conditioning regimes for autoimmune diseases [65]. Another methodic question is whether to use CD34+ selection in autologous HSCT for autoimmune diseases, as already discussed earlier. Over time, graft manipulation was used less frequently in studies for CD, because of the lack of evidence supporting a benefit of this method [27].

So far, allogeneic HSCT has been widely avoided in autoimmune diseases because of its much less attractive risk–benefit ratio. However, a recent pilot study reported higher efficacy compared to autologous HSCT but was stopped due to a fatal infectious complication [50].

### 3.2. Selecting CD Patients for HSCT

Traditionally, HSCT has been considered mainly as a last resort in patients in whom no therapeutic options were left to suppress inflammation. This included cases refractory to every form of approved pharmacologic therapy, as well as novel approaches in phase-three trials. Furthermore, surgical resection of inflamed bowel segments was considered unfeasible in these patients due to the risk of developing major post-surgical complications, e.g., short bowel syndrome. Most patients included in published case series suffered from ileocolonic CD, and upper gastrointestinal tract involvement was reported for some individuals [41]. As patients usually report failure to multiple lines of medical treatment, and previous surgery before HSCT is also considered, there is typically long-standing CD at this point. This is associated with structural damage of the intestine due to fibrosis and scarring, which are not amenable by HSCT. It is therefore necessary to obtain evidence of active ongoing intestinal inflammation prior to the procedure [32]. Long-standing active disease and pre-treatment with multiple immunomodulatory and immunosuppressive medications can also affect the yield of harvested PBSCs [32,41,45]. The question therefore arises if it would be reasonable to use HSCT earlier in the treatment concept of CD to achieve better results, but this approach appears to be limited by the safety profile of this intervention.

In a post hoc analysis of the ASTIC trial [46], shorter disease duration and lower baseline CDAI were associated with steroid-free remission for at least three months after transplantation and factors that favor adverse events (smoking, perianal disease) were confirmed. Interestingly, these factors mirror those reported in studies of more conventional medical approaches to CD, e.g., anti-TNFa antibodies. Due to the small number of cases reported and the negative outcome of the only RCT performed so far, currently no information on potential predictive markers for response to autologous HSCT is available, and evidence-based selection of patients best suited for HSCT is not possible. This corresponds to the lack of predictive biomarkers of response to monoclonal antibodies and targeted small molecules in CD. Based on the mechanism of action of HSCT, one might assume that autologous HSCT would not be able to address monogenic IBD.

In summary, a pragmatic strategy for patient selection might include a history of CD refractory to immunosuppression and biologic therapies, with evidence of ongoing intestinal inflammation not addressable by surgery. To adequately diagnose refractory disease, biologic treatments have to be employed using sufficient doses and time frames [13]. Extensive discussion about therapeutic options with the patient and shared decision making are obligatory. It has been reported that some patients opted for HSCT even when surgical therapy would have been possible, e.g., in cases where proctocolectomy was declined by patients [50]. Furthermore, it is necessary to exclude the presence of infectious complications of CD.

As the therapeutic armamentarium in CD is continually expanding, and new treatment options such as IL23p19 blocking antibodies and Janus kinase inhibitors become available [66], the pool of patients qualifying for HSCT under this strict selection regimen may become even smaller. Nevertheless, it has been estimated that as much as 10% of CD patients are not sufficiently controlled with the currently available therapies [41]. This problem may persist even with the newer drugs, as the number of previous biologic therapies is associated with lower response rates regardless of the mode of action. Therefore, these new options seem to disproportionally benefit patients with forms of IBD responding to therapy [67]. HSCT could be an option for these refractory patients. However, careful consideration of alternative treatment modalities is important. In CD, surgery can be necessary and appropriate [68]. This is especially the case in perianal fistulizing disease, where complete fistula closure by medical therapy alone is not frequently achieved [68]. In addition to fistula drainage and ligation, autologous mesenchymal stem cell transplantation has been suggested for perianal fistula in CD [69]. Small bowel strictures can be addressed by balloon dilatation or stricturoplasty using various surgical techniques [70,71,72]. In contrast to bowel resection, these approaches do not result in bowel loss and therefore do not aggravate the potential risk for small bowel syndrome.

## 4. Future Perspectives and Conclusions

At the time of writing this review, there are three ongoing trials in the United States which are actively recruiting patients for autologous HSCT in CD. Two of these trials are using high-dose immunoablation followed by CD34-selected peripheral blood stem cells (PBSC) in pediatric and adult patients with severe CD (NCT04224558, NCT00692939).

The third ongoing trial has a different, yet very interesting approach: the investigators’ plan is to incorporate the drug vedolizumab after autologous HSCT as maintenance therapy (NCT03219359) to address the low long-term remission rates after HSCT in previous trials which did not include routine application of an anti-inflammatory maintenance therapy directly after HSCT. The monoclonal antibody vedolizumab binds to α4β7 on inflammatory T cells, resulting in gut-selective anti-inflammatory activity [73]. The rationale for using vedolizumab post-transplant is that the investigators expect these T cells to be present in the graft. Thus, their blockage may improve remission rates and may help to maintain remission. The general idea of HSCT in CD is to reprogram the patient’s immune system to its state prior to the autoimmune disorder. Interestingly, previous studies showed that patients post-HSCT regained susceptibility to previously failed medications. Thus, the results of this last-mentioned ongoing trial might help to obtain insight into this immune modulation and concomitant restoration of response, which offers a future therapeutic approach.

As described previously, allogenic HCST exhibits serious and potentially life-threatening complication such as GvHD and infections, as well as associated morbidity. In addition to the low relapse rates in refractory CD, another potential factor favoring allogeneic stem cell grafting over autologous cells is the less toxic conditioning regimen (reduced-intensity conditioning regimen). However, a potentially extended use of immunosuppressive medications limits this fact. In the future, optimal supportive therapy combined with a patient-tailored conditioning regimen could help to improve outcomes in CD patients undergoing this procedure.

Overall, HSCT is a rarely used procedure in CD, with relevant morbidity and mortality. To date, mainly autologous HSCT was investigated in CD patients, although allogenic HSCT provides promising outcome data, but probably imposes a further unfavorable safety profile. As for now, due to the uncertain long-term effects, the low evidence, the lack of a standardized protocol, the questionable risk–benefit ratio, and the increasing availability of new biologics with an eminent safety profile, HSCT should only be considered in highly selected patients who are refractory to medical therapy and who have evidence of ongoing, active intestinal inflammation not addressable by surgery.

## Figures and Tables

**Table 1:** Summary of available evidence for hematopoietic stem cell transplantation in Crohn’s disease. Except for Burt et al., 2020 (allogenic, non-myeloablative hematopoietic stem cell transplantation), all reported studies investigated autologous hematopoietic stem cell transplantation. Included patients in the reported studies are mainly adults, although Burt et al., 2003, Burt et al., 2010, Jauregui-Amezaga et al., 2016, López-García et al., 2017, and Hernanz et al., 2018 also included adolescents with a minimum age of 15 years. The study populations between the different reported trials may overlap in certain manuscripts.

First Author	Year of Publication	Study Design	Included Patients	Conditioning Regimen	CD34+ Selection	Outcomes	Reported Adverse Events during the Transplantation Phase	Relapse Rates	Transplantation-Related Mortality	Length of Follow-Up
Burt et al. [52]	2003	Case series	2	Cy 200 mg/kg + ATG	Yes	2/2 clinical remission, 2/2 endoscopic improvement	Febrile neutropenia (2)	None	None	12 and 15 months
Craig et al. [53]	2003	Case series	4	Cy 200 mg/kg + ATG	Unknown	4/4 clinical remission	Febrile neutropenia (4)	None	None	2 weeks to one year
Scimè et al. [37]	2004	Case report	1	Cy 200 mg/kg + ATG	Yes	1/1 clinical remission, 1/1 endoscopic improvement	*Staphylococcus epidermidis* bacteremia during neutropenia (1)	None	None	5 months
Cassinotti et al. [39]	2007	Phase I/II, prospective	4	CY 200 mg/kg + ATG	No	4/4 clinical remission, 2/3 endoscopic remission at 3 months	Febrile neutropenia (4), perianal abscess (1), pleural and pericardial effusions (1), BK virus-related macrohematuria (1)	1/4	None	11–20 months
Burt et al. [38]	2010	Phase I/II, prospective	24	CY 200 mg/kg + ATG	Yes	clinical relapse-free survival: 91% after 1 year, 19% at 5 years	Febrile neutropenia, bacteremia (6)	15/23	None, 1 accidental death	6 months to 5 years
Hommes et al. [40]	2011	Case series	3 (2 transplanted)	CY 200 mg/kg + ATG	Yes	2/2 clinical and endoscopic improvement at 8 to 10 weeks	Febrile neutropenia, allergic reaction to ATG, bacteremia (1), *C. difficile* infection (1), Rotavirus Infection (2)	2/2	None	5–6 years
Hasselblatt et al. [41]	2012	Phase I/II, prospective	12 (9 transplanted)	CY 200 mg/kg	Yes	4/8 clinical remission after 6 months, 5/9 mucosal healing at 9 months	Febrile neutropenia, bacteremia (2), *C. difficile* infection (2)	7/9	None	6 months to 10 years
Snowden et al. [42]	2014	Retrospective case series	6	CY 200 mg/kg + ATG	2 out of 6	5/6 clinical remissison at 3 months, 5/6 endoscopic remission	Febrile neutropenia, bacteremia, upper gastrointestinal hemorrhage (1)	6/6	None	50–123 months
Hawkey et al. [45]	2015	Phase II, open-label, randomized 1:1	45 (23 transplanted)	CY 200 mg/kg + ATG	No	2/23 sustained disease remission at 1 year	76 serious adverse events (viral infections (9), neutropenic sepsis (8)	20/21	1 (SOS)	One year (primary endpoint)
Jauregui-Amezaga et al. [43]	2016	Phase I/II, prospective	28 (21 transplanted)	CY 200 mg/kg + ATG	No	Not reported	Febrile neutropenia (20), bacteremia (8), septic shock (3), CMV infections (2: one death, one colectomy)	Not reported	1 (CMV infection)	One year (primary endpoint)
Lindsay et al. [46]	2017	ASTIC trial post hoc analysis	40	CY 200 mg/kg + ATG	No	13/34 steroid-free clinical remission, 19/38 endoscopic healing at 1 year	76 serious adverse events in 23 patients	7/38 restarted anti-TNF therapy	1 (SOS)	One year (primary endpoint)
López-García et al. [44]	2017	Phase I/II, prospective	35 (29 transplanted)	CY 200 mg/kg + ATG	No	Clinical and endoscopic relapse-free survival: 61% at one year, 15% at five years	Febrile neutropenia (23), septic shock (1), CMV infections (2: one death, one colectomy)	15/28	1 (CMV infection)	6–60 months
Ruiz et al. [26]	2017	Phase I/II, prospective	14	CY 200 mg/kg + ATG	No	13/14 clinical remission at 30 days	5 identified bacterial infections	1/14	None	30 days
Brierley and Castilla-Llorente et al. [47]	2018	Retrospective survey	82	CY 200 mg/kg + ATG	9 out of 82	51/80 clincial remission at 100 days	Infection within 1 year (22), secondary autoimmune diseases (9), malignancies (5)	60/82	1 (CMV infection), 1 (sepsis at 8 years)	6–174 months
Hernanz et al. [48]	2018	Retrospective case series	7	CY 200 mg/kg + ATG	No	3/7 clinical and endoscopic remission, 1/7 clinical improvement at 6 months	Febrile neutropenia (6), *C. difficile* infection (1)	5/7	None	17–78 months
Burt et al. [50]	2020	Pilot study	9	CY 200 mg/kg + Fludarabine + Alemtuzumab	No	8/8 remission at 5 years	*C. difficile* infection (1), bacteremia (3), chronic limited GvHD (1)	0/8	1 (adenovirus infection)	5 years

CY = cyclophosphamide, ATG = rapid or horse anti-thymocyte globulin, SOS = sinusoidal obstruction syndrome, CMV = cytomegalovirus.

## Data Availability

Not applicable.

## References

[1] Feuerstein J.D., Cheifetz A.S. (2017). Crohn Disease: Epidemiology, Diagnosis, and Management. Mayo Clin. Proc..

[2] Torres J., Mehandru S., Colombel J.F., Peyrin-Biroulet L. (2017). Crohn’s disease. Lancet.

[3] Nambu R., Warner N., Mulder D.J., Kotlarz D., McGovern D.P.B., Cho J., Klein C., Snapper S.B., Griffiths A.M., Iwama I. (2022). A Systematic Review of Monogenic Inflammatory Bowel Disease. Clin. Gastroenterol. Hepatol..

[4] Uhlig H.H., Schwerd T., Koletzko S., Shah N., Kammermeier J., Elkadri A., Ouahed J., Wilson D.C., Travis S.P., Turner D. (2014). The diagnostic approach to monogenic very early onset inflammatory bowel disease. Gastroenterology.

[5] Kaser A., Zeissig S., Blumberg R.S. (2010). Inflammatory bowel disease. Annu. Rev. Immunol..

[6] Cohen N.A., Rubin D.T. (2021). New targets in inflammatory bowel disease therapy: 2021. Curr. Opin. Gastroenterol..

[7] Gade A.K., Douthit N.T., Townsley E. (2020). Medical Management of Crohn’s Disease. Cureus.

[8] Vermeire S., D’Haens G., Baert F., Danese S., Kobayashi T., Loftus E.V., Bhatia S., Agboton C., Rosario M., Chen C. (2022). Efficacy and Safety of Subcutaneous Vedolizumab in Patients With Moderately to Severely Active Crohn’s Disease: Results From the VISIBLE 2 Randomised Trial. J. Crohn’s Colitis.

[9] Feagan B.G., Sandborn W.J., Gasink C., Jacobstein D., Lang Y., Friedman J.R., Blank M.A., Johanns J., Gao L.L., Miao Y. (2016). Ustekinumab as Induction and Maintenance Therapy for Crohn’s Disease. N. Engl. J. Med..

[10] Sandborn W.J., Hanauer S.B., Rutgeerts P., Fedorak R.N., Lukas M., MacIntosh D.G., Panaccione R., Wolf D., Kent J.D., Bittle B. (2007). Adalimumab for maintenance treatment of Crohn’s disease: Results of the CLASSIC II trial. Gut.

[11] Sandborn W.J., Feagan B.G., Loftus E.V., Peyrin-Biroulet L., Van Assche G., D’Haens G., Schreiber S., Colombel J.F., Lewis J.D., Ghosh S. (2020). Efficacy and Safety of Upadacitinib in a Randomized Trial of Patients with Crohn’s Disease. Gastroenterology.

[12] D’Haens G., Panaccione R., Baert F., Bossuyt P., Colombel J.F., Danese S., Dubinsky M., Feagan B.G., Hisamatsu T., Lim A. (2022). Risankizumab as induction therapy for Crohn’s disease: Results from the phase 3 ADVANCE and MOTIVATE induction trials. Lancet.

[13] Torres J., Bonovas S., Doherty G., Kucharzik T., Gisbert J.P., Raine T., Adamina M., Armuzzi A., Bachmann O., Bager P. (2020). ECCO Guidelines on Therapeutics in Crohn’s Disease: Medical Treatment. J. Crohn’s Colitis.

[14] Osgood E.E., Riddle M.C., Mathews T.J. (1939). Aplastic anemia treated with daily transfusions and intravenous marrow; case report. Ann. Intern. Med..

[15] Henig I., Zuckerman T. (2014). Hematopoietic stem cell transplantation-50 years of evolution and future perspectives. Rambam Maimonides Med. J..

[16] Swart J.F., Delemarre E.M., van Wijk F., Boelens J.J., Kuball J., van Laar J.M., Wulffraat N.M. (2017). Haematopoietic stem cell transplantation for autoimmune diseases. Nat. Rev. Rheumatol..

[17] Hügle T., Daikeler T. (2010). Stem cell transplantation for autoimmune diseases. Haematologica.

[18] Oliveira M.C., Elias J.B., Moraes D.A., Simões B.P., Rodrigues M., Ribeiro A.A.F., Piron-Ruiz L., Ruiz M.A., Hamerschlak N. (2021). A review of hematopoietic stem cell transplantation for autoimmune diseases: Multiple sclerosis, systemic sclerosis and Crohn’s disease. Position paper of the Brazilian Society of Bone Marrow Transplantation. Hematol. Transfus. Cell Ther..

[19] Penack O., Peczynski C., Mohty M., Yakoub-Agha I., Styczynski J., Montoto S., Duarte R.F., Kröger N., Schoemans H., Koenecke C. (2020). How much has allogeneic stem cell transplant-related mortality improved since the 1980s? A retrospective analysis from the EBMT. Blood Adv..

[20] Pockley A.G., Lindsay J.O., Foulds G.A., Rutella S., Gribben J.G., Alexander T., Snowden J.A. (2018). Immune Reconstitution After Autologous Hematopoietic Stem Cell Transplantation in Crohn’s Disease: Current Status and Future Directions. A Review on Behalf of the EBMT Autoimmune Diseases Working Party and the Autologous Stem Cell Transplantation In Refractory CD-Low Intensity Therapy Evaluation Study Investigators. Front. Immunol..

[21] Musso M., Porretto F., Crescimanno A., Bondì F., Polizzi V., Scalone R. (2000). Crohn’s disease complicated by relapsed extranodal Hodgkin’s lymphoma: Prolonged complete remission after unmanipulated PBPC autotransplant. Bone Marrow Transplant..

[22] Ditschkowski M., Einsele H., Schwerdtfeger R., Bunjes D., Trenschel R., Beelen D.W., Elmaagacli A.H. (2003). Improvement of inflammatory bowel disease after allogeneic stem-cell transplantation. Transplantation.

[23] Drakos P.E., Nagler A., Or R. (1993). Case of Crohn’s disease in bone marrow transplantation. Am. J. Hematol..

[24] Kashyap A., Forman S.J. (1998). Autologous bone marrow transplantation for non-Hodgkin’s lymphoma resulting in long-term remission of coincidental Crohn’s disease. Br. J. Haematol..

[25] Lopez-Cubero S.O., Sullivan K.M., McDonald G.B. (1998). Course of Crohn’s disease after allogeneic marrow transplantation. Gastroenterology.

[26] Ruiz M.A., Kaiser Junior R.L., Piron-Ruiz L., Peña-Arciniegas T., Saran P.S., De Quadros L.G. (2018). Hematopoietic stem cell transplantation for Crohn’s disease: Gaps, doubts and perspectives. World J. Stem Cells.

[27] Snowden J.A., Panés J., Alexander T., Allez M., Ardizzone S., Dierickx D., Finke J., Hasselblatt P., Hawkey C., Kazmi M. (2018). Autologous Haematopoietic Stem Cell Transplantation (AHSCT) in Severe Crohn’s Disease: A Review on Behalf of ECCO and EBMT. J. Crohn’s Colitis.

[28] El-Ghammaz A.M.S., Ben Matoug R., Elzimaity M., Mostafa N. (2017). Nutritional status of allogeneic hematopoietic stem cell transplantation recipients: Influencing risk factors and impact on survival. Support. Care Cancer.

[29] Lee C.H., Yoon H., Oh D.J., Lee J.M., Choi Y.J., Shin C.M., Park Y.S., Kim N., Lee D.H., Kim J.S. (2020). The prevalence of sarcopenia and its effect on prognosis in patients with Crohn’s disease. Intest. Res..

[30] Afessa B., Abdulai R.M., Kremers W.K., Hogan W.J., Litzow M.R., Peters S.G. (2012). Risk factors and outcome of pulmonary complications after autologous hematopoietic stem cell transplant. Chest.

[31] Blaes A., Konety S., Hurley P. (2016). Cardiovascular Complications of Hematopoietic Stem Cell Transplantation. Curr. Treat. Options Cardiovasc. Med..

[32] Jessop H., Farge D., Saccardi R., Alexander T., Rovira M., Sharrack B., Greco R., Wulffraat N., Moore J., Kazmi M. (2019). General information for patients and carers considering haematopoietic stem cell transplantation (HSCT) for severe autoimmune diseases (ADs): A position statement from the EBMT Autoimmune Diseases Working Party (ADWP), the EBMT Nurses Group, the EBMT Patient, Family and Donor Committee and the Joint Accreditation Committee of ISCT and EBMT (JACIE). Bone Marrow Transplant..

[33] Arnaout K., Patel N., Jain M., El-Amm J., Amro F., Tabbara I.A. (2014). Complications of allogeneic hematopoietic stem cell transplantation. Cancer Investig..

[34] Burt R.K., Fassas A., Snowden J., van Laar J.M., Kozak T., Wulffraat N.M., Nash R.A., Dunbar C.E., Arnold R., Prentice G. (2001). Collection of hematopoietic stem cells from patients with autoimmune diseases. Bone Marrow Transplant..

[35] Statkute L., Verda L., Oyama Y., Traynor A., Villa M., Shook T., Clifton R., Jovanovic B., Satkus J., Loh Y. (2007). Mobilization, harvesting and selection of peripheral blood stem cells in patients with autoimmune diseases undergoing autologous hematopoietic stem cell transplantation. Bone Marrow Transplant..

[36] Vasiliu I.M., Petri M.A., Baer A.N. (2006). Therapy with granulocyte colony-stimulating factor in systemic lupus erythematosus may be associated with severe flares. J. Rheumatol..

[37] Scimè R., Cavallaro A.M., Tringali S., Santoro A., Rizzo A., Montalbano L., Casà A., Cottone M. (2004). Complete clinical remission after high-dose immune suppression and autologous hematopoietic stem cell transplantation in severe Crohn’s disease refractory to immunosuppressive and immunomodulator therapy. Inflamm. Bowel Dis..

[38] Burt R.K., Craig R.M., Milanetti F., Quigley K., Gozdziak P., Bucha J., Testori A., Halverson A., Verda L., de Villiers W.J. (2010). Autologous nonmyeloablative hematopoietic stem cell transplantation in patients with severe anti-TNF refractory Crohn disease: Long-term follow-up. Blood.

[39] Cassinotti A., Annaloro C., Ardizzone S., Onida F., Della Volpe A., Clerici M., Usardi P., Greco S., Maconi G., Porro G.B. (2008). Autologous haematopoietic stem cell transplantation without CD34+ cell selection in refractory Crohn’s disease. Gut.

[40] Hommes D.W., Duijvestein M., Zelinkova Z., Stokkers P.C., Ley M.H., Stoker J., Voermans C., van Oers M.H., Kersten M.J. (2011). Long-term follow-up of autologous hematopoietic stem cell transplantation for severe refractory Crohn’s disease. J. Crohn’s Colitis.

[41] Hasselblatt P., Drognitz K., Potthoff K., Bertz H., Kruis W., Schmidt C., Stallmach A., Schmitt-Graeff A., Finke J., Kreisel W. (2012). Remission of refractory Crohn’s disease by high-dose cyclophosphamide and autologous peripheral blood stem cell transplantation. Aliment. Pharmacol. Ther..

[42] Snowden J.A., Ansari A., Sachchithanantham S., Jackson G., Thompson N., Lobo A., Sanderson J., Kazmi M. (2014). Autologous stem cell transplantation in severe treatment-resistant Crohn’s disease: Long-term follow-up of UK patients treated on compassionate basis. QJM.

[43] Jauregui-Amezaga A., Rovira M., Marín P., Salas A., Pinó-Donnay S., Feu F., Elizalde J.I., Fernández-Avilés F., Martínez C., Gutiérrez G. (2016). Improving safety of autologous haematopoietic stem cell transplantation in patients with Crohn’s disease. Gut.

[44] López-García A., Rovira M., Jauregui-Amezaga A., Marín P., Barastegui R., Salas A., Ribas V., Feu F., Elizalde J.I., Fernández-Avilés F. (2017). Autologous Haematopoietic Stem Cell Transplantation for Refractory Crohn’s Disease: Efficacy in a Single-Centre Cohort. J. Crohn’s Colitis.

[45] Hawkey C.J., Allez M., Clark M.M., Labopin M., Lindsay J.O., Ricart E., Rogler G., Rovira M., Satsangi J., Danese S. (2015). Autologous Hematopoetic Stem Cell Transplantation for Refractory Crohn Disease: A Randomized Clinical Trial. JAMA.

[46] Lindsay J.O., Allez M., Clark M., Labopin M., Ricart E., Rogler G., Rovira M., Satsangi J., Farge D., Hawkey C.J. (2017). Autologous stem-cell transplantation in treatment-refractory Crohn’s disease: An analysis of pooled data from the ASTIC trial. Lancet Gastroenterol. Hepatol..

[47] Brierley C.K., Castilla-Llorente C., Labopin M., Badoglio M., Rovira M., Ricart E., Dierickx D., Vermeire S., Hasselblatt P., Finke J. (2018). Autologous Haematopoietic Stem Cell Transplantation for Crohn’s Disease: A Retrospective Survey of Long-term Outcomes From the European Society for Blood and Marrow Transplantation. J. Crohn’s Colitis.

[48] Hernanz N., Sierra M., Volpato N., Núñez-Gómez L., Mesonero F., Herrera-Puente P., García-Gutiérrez V., Albillos A., López-San Román A. (2019). Autologous haematopoietic stem cell transplantation in refractory Crohn’s disease: Experience in our centre. Gastroenterol. Hepatol..

[49] Snowden J.A., Saccardi R., Allez M., Ardizzone S., Arnold R., Cervera R., Denton C., Hawkey C., Labopin M., Mancardi G. (2012). Haematopoietic SCT in severe autoimmune diseases: Updated guidelines of the European Group for Blood and Marrow Transplantation. Bone Marrow Transplant..

[50] Burt R.K., Craig R., Yun L., Halverson A., Quigley K., Arnautovic I., Han X. (2020). A pilot feasibility study of non-myeloablative allogeneic hematopoietic stem cell transplantation for refractory Crohn Disease. Bone Marrow Transplant..

[51] Kelsey P.J., Oliveira M.C., Badoglio M., Sharrack B., Farge D., Snowden J.A. (2016). Haematopoietic stem cell transplantation in autoimmune diseases: From basic science to clinical practice. Curr. Res. Transl. Med..

[52] Burt R.K., Traynor A., Oyama Y., Craig R. (2003). High-dose immune suppression and autologous hematopoietic stem cell transplantation in refractory Crohn disease. Blood.

[53] Craig R.M., Traynor A., Oyama Y., Burt R.K. (2003). Hematopoietic stem cell transplantation for severe Crohn’s disease. Bone Marrow Transplant..

[54] El-Nakeep S., Shawky A., Abbas S.F., Abdel Latif O. (2022). Stem cell transplantation for induction of remission in medically refractory Crohn’s disease. Cochrane Database Syst. Rev..

[55] Mikulska M., Del Bono V., Bruzzi P., Raiola A.M., Gualandi F., Van Lint M.T., Bacigalupo A., Viscoli C. (2012). Mortality after bloodstream infections in allogeneic haematopoietic stem cell transplant (HSCT) recipients. Infection.

[56] Busmail A., Penumetcha S.S., Ahluwalia S., Irfan R., Khan S.A., Rohit Reddy S., Vasquez Lopez M.E., Zahid M., Mohammed L. (2022). A Systematic Review on Pulmonary Complications Secondary to Hematopoietic Stem Cell Transplantation. Cureus.

[57] Giménez E., Torres I., Albert E., Piñana J.L., Hernández-Boluda J.C., Solano C., Navarro D. (2019). Cytomegalovirus (CMV) infection and risk of mortality in allogeneic hematopoietic stem cell transplantation (Allo-HSCT): A systematic review, meta-analysis, and meta-regression analysis. Am. J. Transplant..

[58] Tanaka Y., Kurosawa S., Tajima K., Tanaka T., Ito R., Inoue Y., Okinaka K., Inamoto Y., Fuji S., Kim S.W. (2016). Analysis of non-relapse mortality and causes of death over 15 years following allogeneic hematopoietic stem cell transplantation. Bone Marrow Transplant..

[59] Sorror M.L., Maris M.B., Storb R., Baron F., Sandmaier B.M., Maloney D.G., Storer B. (2005). Hematopoietic cell transplantation (HCT)-specific comorbidity index: A new tool for risk assessment before allogeneic HCT. Blood.

[60] Petersdorf E.W., Stevenson P., Bengtsson M., De Santis D., Dubois V., Gooley T., Horowitz M., Hsu K., Madrigal J.A., Malkki M. (2020). HLA-B leader and survivorship after HLA-mismatched unrelated donor transplantation. Blood.

[61] Fuchs E.J., McCurdy S.R., Solomon S.R., Wang T., Herr M.R., Modi D., Grunwald M.R., Nishihori T., Kuxhausen M., Fingerson S. (2022). HLA informs risk predictions after haploidentical stem cell transplantation with posttransplantation cyclophosphamide. Blood.

[62] Burisch J., Kiudelis G., Kupcinskas L., Kievit H.A.L., Andersen K.W., Andersen V., Salupere R., Pedersen N., Kjeldsen J., D’Incà R. (2019). Natural disease course of Crohn’s disease during the first 5 years after diagnosis in a European population-based inception cohort: An Epi-IBD study. Gut.

[63] Sandborn W.J., D’Haens G.R., Reinisch W., Panés J., Chan D., Gonzalez S., Weisel K., Germinaro M., Frustaci M.E., Yang Z. (2022). Guselkumab for the Treatment of Crohn’s Disease: Induction Results From the Phase 2 GALAXI-1 Study. Gastroenterology.

[64] Mohty M. (2007). Mechanisms of action of antithymocyte globulin: T-cell depletion and beyond. Leukemia.

[65] Loh Y., Oyama Y., Statkute L., Quigley K., Yaung K., Gonda E., Barr W., Jovanovic B., Craig R., Stefoski D. (2007). Development of a secondary autoimmune disorder after hematopoietic stem cell transplantation for autoimmune diseases: Role of conditioning regimen used. Blood.

[66] Grossberg L.B., Papamichael K., Cheifetz A.S. (2022). Review article: Emerging drug therapies in inflammatory bowel disease. Aliment. Pharmacol. Ther..

[67] Danese S., Parigi T.L., Peyrin-Biroulet L., Ghosh S. (2021). Defining difficult-to-treat inflammatory bowel disease: Why and how. Lancet Gastroenterol. Hepatol..

[68] Adamina M., Bonovas S., Raine T., Spinelli A., Warusavitarne J., Armuzzi A., Bachmann O., Bager P., Biancone L., Bokemeyer B. (2020). ECCO Guidelines on Therapeutics in Crohn’s Disease: Surgical Treatment. J. Crohn’s Colitis.

[69] Lee W.Y., Park K.J., Cho Y.B., Yoon S.N., Song K.H., Kim D.S., Jung S.H., Kim M., Yoo H.W., Kim I. (2013). Autologous adipose tissue-derived stem cells treatment demonstrated favorable and sustainable therapeutic effect for Crohn’s fistula. Stem Cells.

[70] Bettenworth D., Gustavsson A., Atreja A., Lopez R., Tysk C., van Assche G., Rieder F. (2017). A Pooled Analysis of Efficacy, Safety, and Long-term Outcome of Endoscopic Balloon Dilation Therapy for Patients with Stricturing Crohn’s Disease. Inflamm. Bowel Dis..

[71] Ozuner G., Fazio V.W., Lavery I.C., Milsom J.W., Strong S.A. (1996). Reoperative rates for Crohn’s disease following strictureplasty. Long-term analysis. Dis. Colon Rectum.

[72] Bellolio F., Cohen Z., MacRae H.M., O’Connor B.I., Victor J.C., Huang H., McLeod R.S. (2012). Strictureplasty in selected Crohn’s disease patients results in acceptable long-term outcome. Dis. Colon Rectum.

[73] Fedyk E.R., Wyant T., Yang L.L., Csizmadia V., Burke K., Yang H., Kadambi V.J. (2012). Exclusive antagonism of the α4 β7 integrin by vedolizumab confirms the gut-selectivity of this pathway in primates. Inflamm. Bowel Dis..

